# From Diagnosis Forward: How Early Information Shapes the Family Caregiving Experience in Dementia

**DOI:** 10.1093/geroni/igaf122.3941

**Published:** 2025-12-31

**Authors:** Tomoko Wakui, Tsuyoshi Okamura, Kae Ito, Takumi Hirata, Tomoyuki Yabuki

**Affiliations:** Tokyo Metropolitan Institute for Geriatrics and Gerontology, Tokyo, Tokyo, Japan; Tokyo Metropolitan Institute for Geriatrics and Gerontology, Itabashi, Tokyo, Japan; Tokyo Metropolitan Institute for Geriatrics and Gerontology, Itabashi, Tokyo, Japan; Tokyo Metropolitan Institute of Gerontology, Itabashi, Tokyo, Japan; University of Kochi, Kochi, Kochi, Japan

## Abstract

Beyond helping individuals and their families come to terms with a dementia diagnosis, appropriate informational support at the time of diagnosis may influence how they manage life with dementia in the years that follow. This study aimed to examine the impact of informational support provided at the time of diagnosis on the current caregiving burden experienced by family members. A self-administered survey was conducted in January 2025 with 164 family caregivers of individuals diagnosed at dementia medical centers and by certified dementia support physicians across Japan. The primary outcome was caregiver burden, assessed using the short version of the Japanese Zarit Caregiving Inventory (J-ZBI_8). Regression analyses controlled for care recipient characteristics, caregiver demographics, and caregiving context, while examining associations between nine types of informational support received at diagnosis and current caregiver burden. Caregivers most frequently reported receiving information about the disease itself (72.0%), long-term care services and Community General Support Centers (58.5%), and local medical institutions (37.2%). Practical information on legal, financial, or employment-related matters was less commonly provided. Among all types of support, only dementia-related information (B = –5.07, p < .001) and information about local medical institutions (B = –2.93, p = .02) were significantly associated with lower caregiver burden. These findings indicate that early, focused informational support—particularly regarding the disease and access to local dementia-specialized clinics—may enhance caregivers’ preparedness and ease anxiety about managing future care needs.

